# Identifying Self-Reported Humor Style as a Potential New Direction in Cognitive Health: A Pilot Study

**DOI:** 10.1093/geroni/igaf122.2050

**Published:** 2025-12-31

**Authors:** Nikki-Anne Wilson, Moyra E Mortby, Justine M Gatt, Fiona Kumfor, Jill Bennett, Henry Brodaty, Kaarin J Anstey

**Affiliations:** University of New South Wales Sydney/Neuroscience Research Australia, Kensington, New South Wales, Australia; University of New South Wales Sydney, Kensington, New South Wales, Australia; Neuroscience Research Australia, Randwick, New South Wales, Australia; The University of Sydney, Camperdown, New South Wales, Australia; University of New South Wales Sydney, Kensington, New South Wales, Australia; University of New South Wales Sydney, Kensington, New South Wales, Australia; University of New South Wales Sydney, Kensington, New South Wales, Australia

## Abstract

Humor is a central aspect of social communication yet little research has explored how its use in everyday life may relate to cognitive performance in older adults. Building on extant literature investigating humor styles and psychosocial health, we examine the association between positive (affiliative, self-enhancing), and negative (self-defeating, aggressive) humor styles and cognitive function in a cross-sectional pilot study. Ninety-five older adults (90% White; 84% culturally Australian, mean age 76 years) completed the Humor Styles Questionnaire and an online cognitive battery including Paired Associates Learning (PAL), Pattern Recognition Memory (PRM), and Spatial Working Memory (SWM) tasks. Multiple linear regression models controlled for age, gender, culture, education, and depressive symptoms. Higher positive humor was significantly associated with better episodic memory (β = 0.30, p =.020). For negative humor style, a significant gender by negative humor interaction (p =.013) showed more negative humor was associated with poorer episodic memory in males (β = -0.43, p =.030) but a trend towards better performance in females (β = 0.23, p =.079). Both males and females showed an association between higher negative humor and better executive function (SWM, β = -2.97, p =.014), however, the significance of this relationship weakened in upper ages (69 years, β = -0.44, p ≤.001; 80 years, β = 0.04, p =.795). We show that self-reported humor style is significantly associated with domain specific cognitive processes. This has important implications for understanding the ways humor use may reflect broader cognitive performance and establishing new directions in cognitive health research.

